# Sustained virological response and metabolic risk factors are associated with mortality in patients with chronic hepatitis C

**DOI:** 10.1371/journal.pone.0208858

**Published:** 2019-01-09

**Authors:** Yi-Hao Yen, Kwong-Ming Kee, Chien-Hung Chen, Tsung-Hui Hu, Sheng-Nan Lu, Jing-Houng Wang, Chao-Hung Hung

**Affiliations:** Division of Hepatogastroenterology, Department of Internal Medicine, Kaohsiung Chang Gung Memorial Hospital and Chang Gung University College of Medicine, Kaohsiung, Taiwan; Nihon University School of Medicine, JAPAN

## Abstract

**Background and aim:**

Previous studies have reported that sustained virological response (SVR) to interferon-based treatment reduces the risk of mortality in chronic hepatitis C (CHC) patients, mainly in cirrhotic patients. A population-based study reported that metabolic risk factors increase the risk of mortality in CHC patients. We aim to investigate the association between SVR, metabolic risk factors and mortality in CHC patients with and without advanced fibrosis.

**Methods:**

We collected data from 1452 CHC patients who underwent interferon-based therapy. All patients underwent liver biopsy prior to therapy. Mild fibrosis was defined as a modified Knodell score of 0–2, while advanced fibrosis was defined as a score of 3–4.

**Results:**

1452 patients were followed up for a median (IQR) of 6.6 (4.2–9.4) years, 1124 patients (77.4%) achieved SVR, 619 patients (42.6%) were advanced fibrosis. 14 patients with mild fibrosis and 55 patients with advanced fibrosis died during follow-up period. According to multivariate Cox regression analyses, SVR reduced the risks of all-cause mortality (HR, 0.21; 95% CI, 0.12–0.37; *P*<0.001), liver-related mortality (HR, 0.19; 95% CI, 0.10–0.38; *P* < .001), and non-liver-related mortality (HR, 0.26; 95% CI, 0.10–0.71; *P* = 0.009) in the patients with advanced fibrosis. SVR also reduced the risk of liver-related mortality (HR, 0.09; 95% CI, 0.01–0.60; P = 0.013) in the patients with mild fibrosis. Anti-hypertensive treatment increased the risks of all-cause mortality (HR, 6.1; 95% CI: 1.66–22.54; P = 0.006) and liver-related mortality (HR, 12.3; 95% CI: 1.4–108.5; P = 0.02) in the patients with mild fibrosis.

**Conclusion:**

SVR and metabolic risk factors are associated with mortality in CHC patients given interferon-based treatment.

## Introduction

Chronic hepatitis C virus (HCV) infection is a major cause of cirrhosis, end-stage liver disease and hepatocellular carcinoma (HCC) [1]. Chronic HCV infection is associated with extrahepatic manifestations, including non-Hodgkin lymphoma, cardiovascular disease and type 2 diabetes mellitus (DM) [2]. Extrahepatic manifestations of HCV may be associated with non-liver-related mortality in chronic HCV-infected patients. Interferon-based therapy clearly reduces the rate of liver-related mortality in patients with chronic HCV infection [3]. Recently, Nahon, et al reported that sustained virological response (SVR) reduces non-liver-related mortality in chronic hepatitis C (CHC) patients with cirrhosis [4]. Therefore, SVR may be associated with the resolution of extrahepatic manifestations, which could in turn explain the decrease in non-liver-related mortality. A population-based study reported that metabolic risk factors increased the risk of liver and non-liver-related mortality in a CHC cohort [5].

Direct-acting antivirals (DAAs) have led to SVR in most patients, including patients with old age, comorbidities and decompensated liver cirrhosis [6]. However, because a full understanding of the clinical benefits of DAAs will require long-term follow-up evaluations, we currently can only rely on long-term results obtained in patients treated by interferon-based regimens to evaluate whether SVR reduces mortality in chronic HCV-infected patients.

In the present large-sample study of patients with baseline histological evaluation of liver fibrosis prior to interferon-based therapy, we analyzed the association of SVR and metabolic risk factors with mortality, including all-cause, liver and non-liver-related mortality, in patients with mild and advanced fibrosis.

## Materials and methods

### Patients

**Fig 1:**
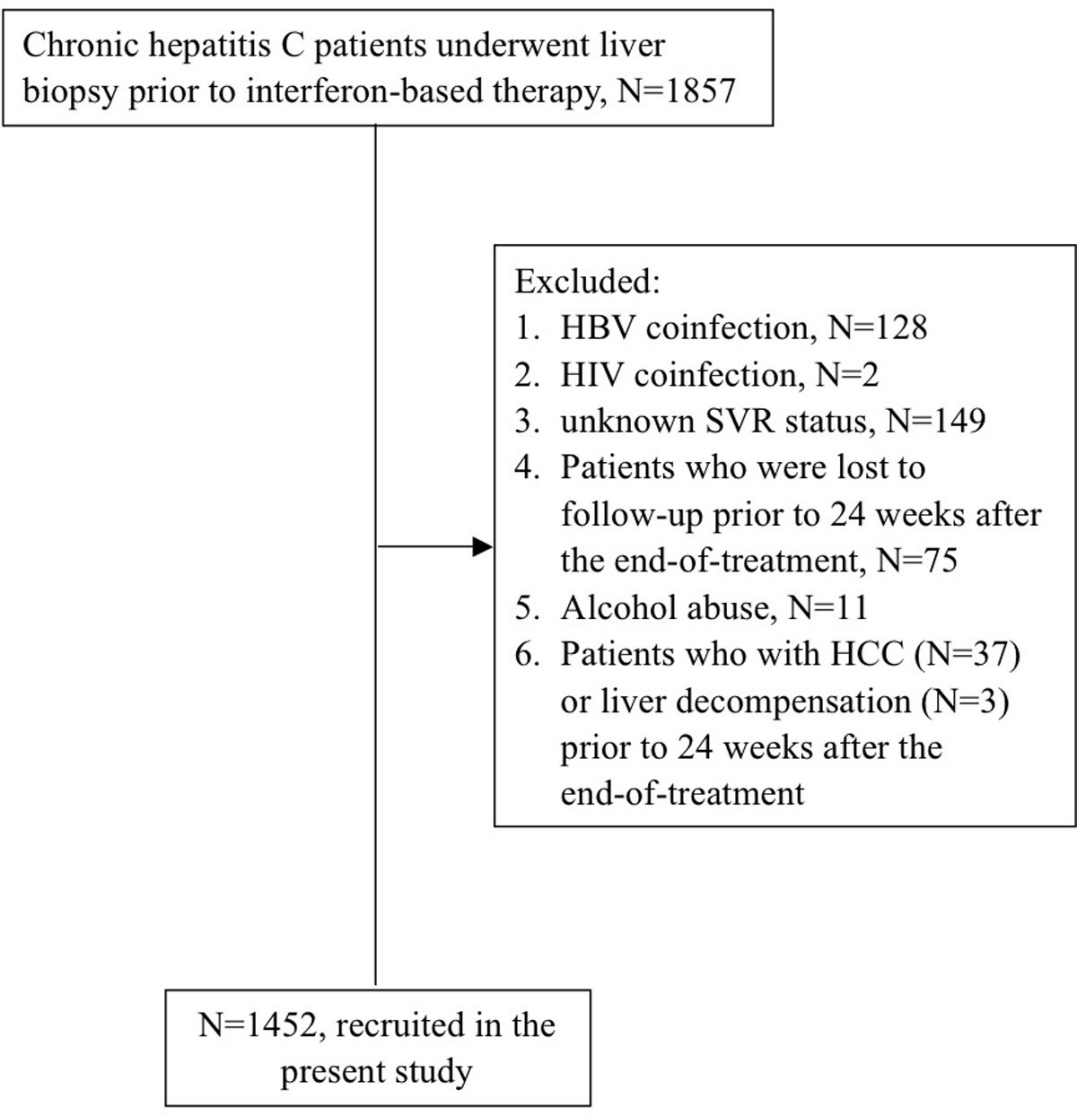
The inclusion and exclusion of potential subjects for this study.

This retrospective study included consecutive adult patients with chronic HCV infection who had undergone liver biopsy prior to interferon-based therapy at Kaohsiung Chang Gung Memorial Hospital from April 1999 to October 2011. Patients with the following conditions were excluded from the study: hepatitis B virus (HBV) or human immunodeficiency virus (HIV) infection, alcohol abuse, and HCC or liver decompensation diagnosed prior to 24 weeks after the end of treatment. Furthermore, patients who were lost to follow-up prior to 24 weeks after the end of treatment and patients with unknown SVR status were also excluded (Fig 1).

All the procedures used in the study were in accordance with the ethical standards of the responsible committees on human experimentation (institutional and national) and with the Helsinki Declaration of 1975, as revised in 2008. This study was approved by the Institutional Review Board of Kaohsiung Chang Gung Memorial Hospital (IRB number: 201601042B0C501). The requirement for informed consent was waived by the IRB. The data were analyzed anonymously.

DM was identified based on diagnoses documented in medical records, a serum fasting glucose level > 126 mg/dL, or the use of antidiabetic drugs [7]. Alcohol abuse was identified according to statements from the treating physician indicating its diagnosis in the medical records. However, tobacco consumption, alcohol intake and injection drug use by patients were not routinely recorded by every treating physician.

The date of 24 weeks after the end of treatment for a given patient was defined as the start of the follow-up period for that patient. The end of the follow-up period was defined as the date of the final visit for those patients who remained alive and as the date of death for those patients who died during follow-up. For analysis, the reference date for analysis was December 31, 2014. The median (IQR) follow-up duration was 6.57 (4.16–9.35) years.

### Liver histology evaluation

The degree of liver fibrosis in each patient was graded and staged according to the modified Knodell histology index [8]. The degree of liver necroinflammation in each patient was calculated by Histology Activity Index scores [9]. The presence of steatosis was defined as steatosis >5% of hepatocytes with fatty change [10].

### Treatment and follow-up evaluation

All the patients were treated with interferon and ribavirin. SVR was defined as undetectable HCV RNA at follow-up week 24 [11]. All the patients underwent HCC surveillance using ultrasound and alpha-fetoprotein (AFP) every 6 months [12]. Any patient with a new lesion observed via ultrasound was further evaluated with contrast-enhanced computed tomography, magnetic resonance imaging, or liver biopsy. The diagnosis of HCC was based on recommended criteria [13, 14].

### Clinical outcome measures

The clinical outcome measures of the study were all-cause mortality, liver-related and non-liver-related mortality. Liver transplantation events and liver-related mortality were analyzed as a combined end point (liver-related mortality). Patients who underwent liver transplantation were censored at the date of transplantation for analysis. Death caused by liver failure or HCC was considered liver-related. Death due to extrahepatic malignancy, bacterial infection, cerebrovascular or cardiovascular events, or other causes was considered non-liver-related. The definition of liver failure included ascites confirmed by ultrasonography, overt hepatic encephalopathy, or bleeding varices.

### Statistical analysis

Baseline characteristics and clinical variables were summarized as mean ± standard deviation, median (interquartile range), or percentage. The chi-square test and independent two-sample t-test were used to assess the significance of differences in distributions. The associations among SVR, metabolic risk factors and mortality were estimated with the Cox proportional hazards regression method. Deceased patients were censored at the time of death. Cumulative incidence and survival curves according to SVR status and metabolic risk factors were built using the Kaplan–Meier method. A comparison of incidence and survival curves according to SVR status and metabolic risk factors were conducted with univariate Cox regression analyses. A P-value of less than 0.05 was considered statistically significant. All analyses were performed using the Stata version 11.0. (StataCorp. 2009, Stata 11 Base Reference Manual, College Station, TX: Stata Press).

## Results

### Study population

The inclusion and exclusion of potential subjects for this study are depicted in Fig 1. Among 1857 screened CHC patients, 1452 (78.3%) were subsequently included. The demographic and clinical characteristics of all the included patients are shown in Table 1. The average baseline age of the patients was 53.9±11.3 years, 756 of the patients (52.1%) were men, and 1124 of the patients (77.4%) achieved SVR. The modified Knodell fibrosis score was 3–4 in 619 patients (42.6%). As expected, the patients without SVR were older, and the proportion of patients with advanced fibrosis and genotype 1 was higher (Table 1). During the entire study period, 37 of the patients without SVR died (including 5 patients with mild fibrosis and 32 patients with advanced fibrosis), and 32 of the patients with SVR died (including 9 patients with mild fibrosis and 23 patients with advanced fibrosis).

**Table 1:** Baseline Characteristics According to Treatment Response in all patients.

Characteristics	Overall (N = 1452)	With SVR (N = 1124)	Without SVR (N = 328)	*P*
Age(yr)	53.9 ± 11.3	53.1 ± 11.1	56.8± 11.3	< 0.001
Male	756 (52.1%)	607 (54.0%)	149 (45.4%)	0.006
Anti-hypertensive treatment	192 (13.2%)	148 (13.2%)	44 (13.4%)	0.91
Anti-lipid treatment	66 (4.6%)	47 (4.2%)	19 (5.8%)	0.22
DM	176 (12.1%)	133 (11.8%)	43 (13.1%)	0.53
BMI (kg/m^2^)				0.33
< 24	594 (45.8%)	473 (46.8%)	121 (42.0%)	
24–27	412 (31.7%)	316 (31.3%)	96 (33.3%)	
>27	292 (22.5%)	221 (21.9%)	71 (24.7%)	
Platelet count <150 (10^9^/L)	577 (40.1%)	405 (36.3%)	172 (53.4%)	< 0.001
AST (IU/L)	91 (62–136)	92 (63–135)	89 (60–137)	0.55
ALT (IU/L)	133 (91–205)	139 (94–215)	118 (86–178)	<0.001
Bilirubin (mg/dL)	0.97 ± 0.41	0.95 ± 0.39	1.03 ± 0.49	0.001
Fibrosis score				< 0.001
0–2	833 (57.4%)	686 (61.0%)	147 (44.8%)	
3–4	619 (42.6%)	438 (39.0%)	181 (55.2%)	
Necroinflammation score				0.81
<9	998 (69.0%)	770 (68.8%)	228 (69.5%)	
≥9	449 (31.0%)	349 (31.2%)	100 (30.5%)	
Genotype				< 0.001
1	589 (40.6%)	386 (34.3%)	203 (61.2%)	
2	695 (47.9%)	623 (55.4%)	72 (22.0%)	
Mixed	56 (3.9%)	45 (4.0%)	11 (3.4%)	
others	112 (7.7%)	70 (6.2%)	42 (12.8%)	
Steatosis >5%	399 (27.5%)	318 (28.3%)	81 (24.8%)	0.21

Data were expressed as mean ± SD or median (interquantile). SVR, sustained virological response; DM, diabetes mellitus; BMI, body mass index; AST, Aspartate Aminotransferase; ALT, Alanine Aminotransferase; Patients were categorized as normal weight or underweight (<24 kg/m^2^), overweight (24–27 kg/m^2^), or obese (>27 kg/m^2^) according to the definition of the Health Promotion Administration of the Ministry of Health and Welfare in Taiwan [15].

### All-cause mortality in all patients

**Fig 2:**
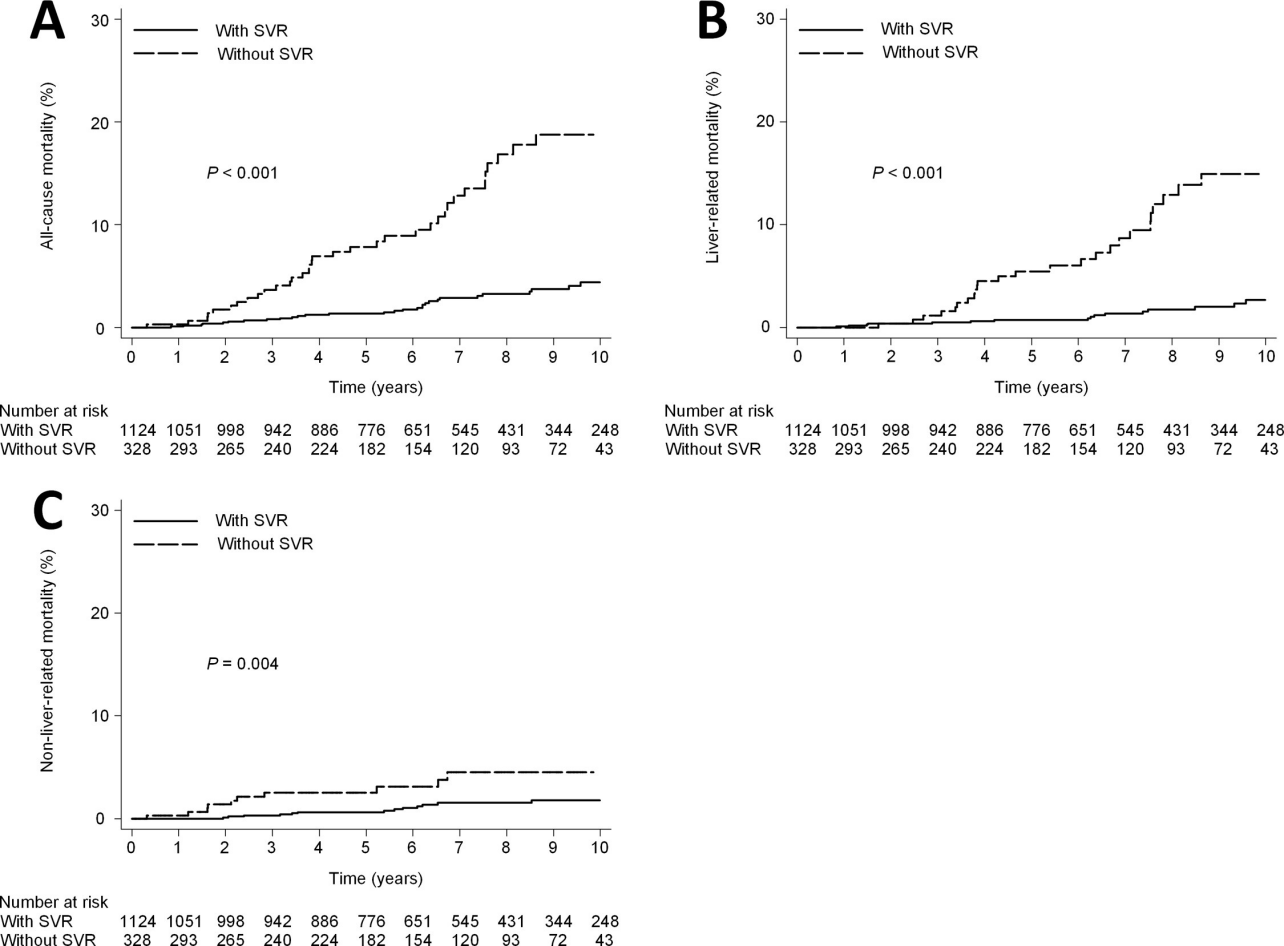
Mortality according to SVR in all patients. (A) all-cause mortality (10- year mortality rate, 4.4% vs 18.8%; HR, 0.21; 95% CI, 0.13–0.33; P<0.001). (B) liver-related mortality (10- year mortality rate, 2.7% vs 15.0%; HR, 0.16; 95% CI, 0.09–0.29; P<0.001). (C) non-liver-related mortality (10- year mortality rate, 1.8% vs 4.5%; HR, 0.31; 95% CI, 0.14–0.69; P = 0.007).

There was a significant difference in the cumulative 10- year mortality rate between the patients with SVR (4.4%; 95% CI, 3.0%-6.5%) and those without SVR (18.8%; 95% CI, 13.5%-25.8%; *P*<0.001) (Fig 2A). Cox proportional hazards regression analysis showed that SVR was associated with a statistically significant reduction in the overall hazard of death (adjusted hazard ratio [HR], 0.24; 95% CI, 0.14–0.38; *P*<0.001). Other baseline factors found to be significantly associated with all-cause mortality in multivariate analysis were anti-hypertensive treatment (adjusted HR, 1.9; 95% CI, 1.0–3.6, P = 0.04), advanced fibrosis, age > 60 years and male gender (Table 2).

**Table 2:** Features Associated With Mortality in All Patients (N = 1452) According to Cox Proportional Hazards Model:Results of Multivariate Analyses.

Variables	comparison		All-cause			Liver-related			Non-liver-related	
		HR	95% CI	*P*	HR	95% CI	*P*	HR	95% CI	*P*
DM	Yes vs no	1.48	0.81–2.71	0.21	0.86	0.36–2.09	0.74	2.84	1.14–7.1	0.03
Anti-lipid treatment	Yes vs no	0.34	0.05–2.55	0.30	0.61	0.08–4.70	0.64	-		
Anti-hypertensive treatment	Yes vs no	1.92	1.03–3.60	0.04	1.38	0.59–3.24	0.46	2.60	0.98–6.88	0.05
Fibrosis score	3–4 vs 0–2	4.54	2.48–8.29	<0.001	7.75	3.22–18.67	<0.001	2.52	1.06–5.99	0.04
SVR	Yes vs no	0.24	0.14–0.38	<0.001	0.17	0.09–0.32	<0.001	0.41	0.18–0.92	0.03
Age (yr)	≥60 vs <60	1.67	1.02–2.72	0.04	1.39	0.75–2.57	0.29	2.14	0.94–4.89	0.07
Sex	Male vs female	1.81	1.11–2.96	0.02	1.92	1.04–3.53	0.04	1.60	0.7–3.65	0.26
Obesity	BMI> vs ≤27 (kg/m^2^)	1.01	0.56–1.84	0.96	1.23	0.61–2.50	0.56	0.67	0.22–2.05	0.48
Steatosis >5%	Yes vs no	0.77	0.43–1.39	0.39	0.99	0.50–1.98	0.99	0.37	0.11–1.26	0.11

DM, diabetes mellitus; SVR, sustained virological response; BMI, body mass index; Patients were categorized as obese (>27 kg/m^2^) according to the definition of the Health Promotion Administration of the Ministry of Health and Welfare in Taiwan [15].

### Liver-related mortality in all patients

The 10-year cumulative incidence risk of liver-related mortality was 2.7% [95% CI, 1.6%-4.5%] in the patients with SVR and 15.0% [95% CI, 10.1%-21.9%] in the patients without SVR (*P*<0.001) (Fig 2B). Cox proportional hazards regression analysis showed that SVR was associated with a statistically significant reduction in the hazard of liver-related-mortality (adjusted HR, 0.17; 95% CI, 0.09–0.32; *P*<0.001). Other baseline factors found to be significantly associated with liver-related mortality in multivariate analysis were advanced fibrosis and male gender (Table 2).

### Non-liver-related mortality in all patients

**Fig 3:**
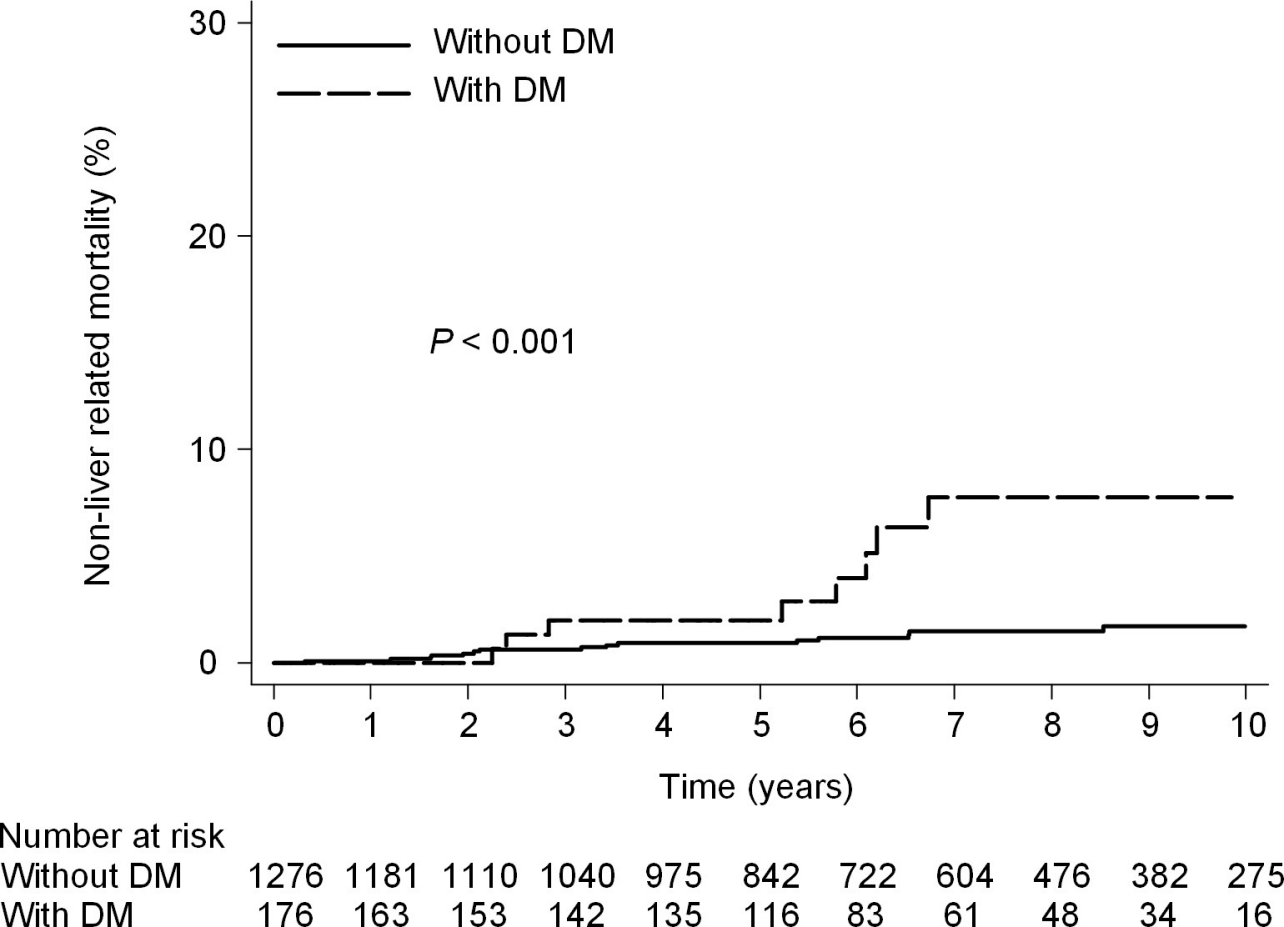
Non-liver-related mortality according to DM. (10- year mortality rate, 1.7% vs 7.7%; HR, 4.8; 95% CI, 2.1–11.0; P<0.001).

The 10-year cumulative incidence risk of non-liver-related mortality was 1.8% [95% CI, 1.0%-3.1%] in the patients with SVR and 4.5% [95% CI, 2.4%-8.5%] in the patients without SVR (*P* = 0.004) (Fig 2C). Cox proportional hazards regression analysis showed that SVR was associated with a statistically significant reduction in the hazard of non-liver-related mortality (adjusted HR, 0.41; 95% CI, 0.18–0.92; *P* = 0.03). Other baseline factors found to be significantly associated with non-liver-related mortality in multivariate analysis were DM (adjusted HR, 2.8; 95% CI, 1.1–7.1; P = 0.03) and advanced fibrosis (Table 2). The 10-year cumulative incidence risk of non-liver-related mortality was 1.7% [95% CI, 1.0%-2.9%] in the patients without DM and 7.7% [95% CI 3.8%-15.3%] in the patients with DM (*P*<0.001) (Fig 3)

### Baseline characteristics of patients with mild fibrosis

There were 833 patients with mild fibrosis, and 686 of these patients (82.4%) achieved SVR. As expected, the non-SVR patients were older, on average, and included a larger proportion of patients with genotype 1 than did the patients who achieved SVR (Table 3). Causes of death are also shown in Table 3.

**Table 3:** Baseline Characteristics According to Treatment Response in mild fibrotic patients.

Characteristics	Overall (N = 833)	With SVR (N = 686)	Without SVR (N = 147)	*P*
Age	51.3 ± 11.8	50.9 ± 11.4	53.3 ± 12.9	0.02
Male	462 (55.5%)	385 (56.1%)	77 (52.4%)	0.41
Anti-hypertension treatment	103 (12.4%)	86 (12.5%)	17 (11.6%)	0.75
Anti-lipid treatment	52 (6.2%)	37 (5.4%)	15 (10.2%)	0.03
DM	90 (10.8%)	70 (10.2%)	20 (13.6%)	0.23
BMI (kg/m^2^)				0.71
< 24	378 (50.5%)	314 (50.7%)	64 (49.6%)	
24–27	216 (28.8%)	181 (29.2%)	35 (27.1%)	
>27	155 (20.7%)	125 (20.2%)	30 (23.3%)	
Platelet count <150 (10^9^/L)	193 (23.5%)	157 (41.3%)	36 (25.2%)	0.59
AST (IU/L)	78 (52–119)	81 (53–121)	71 (46–96)	0.008
ALT (IU/L)	124 (84–205)	128.5 (86–213)	107.5 (70–150)	0.002
Bilirubin (mg/dL)	0.9 ± 0.37	0.9 ± 0.36	0.9 ± 0.42	0.96
Necroinflammation score				0.08
<9	661 (79.7%)	536 (78.6%)	125 (85.0%)	
≥9	168 (20.3%)	146 (21.4%)	22 (15.0%)	
Genotype				<0.001
1	332 (42.6%)	235 (34.3%)	97 (66.0%)	
2	417 (53.5%)	385 (56.1%)	32 (21.8%)	
Mixed	31 (4.0%)	27 (3.9%)	4 (2.7%)	
others	53 (6.4%)	39 (5.7%)	14 (9.5%)	
Steatosis >5%	217 (26.1%)	176 (25.7%)	41 (28.1%)	0.08
Liver-related mortality				
		Liver failure,N = 1 HCC, N = 1	Liver failure, N = 3 HCC, N = 1	
Non–liver-related mortality				
		Heart failure, N = 1 Traumatic intracranial hemorrhage, N = 1 Pancreatic cancer, N = 1 Colon cancer, N = 1 Parotid gland cancer, N = 1 Bacterial infection, N = 1 Acute myocardial infarction, N = 1	Common bile duct cholangiocarcionoma, N = 1	

Data were expressed as mean ± SD or median (interquantile). DM, diabetes mellitus; BMI, body mass index; AST, Aspartate Aminotransferase; ALT, Alanine Aminotransferase; HCC, hepatocellular carcinoma. Patients were categorized as normal weight or underweight (<24 kg/m^2^), overweight (24–27 kg/m^2^), or obese (>27 kg/m^2^) according to the definition of the Health Promotion Administration of the Ministry of Health and Welfare in Taiwan [15].

### All-cause mortality in mild fibrotic patients

**Fig 4:**
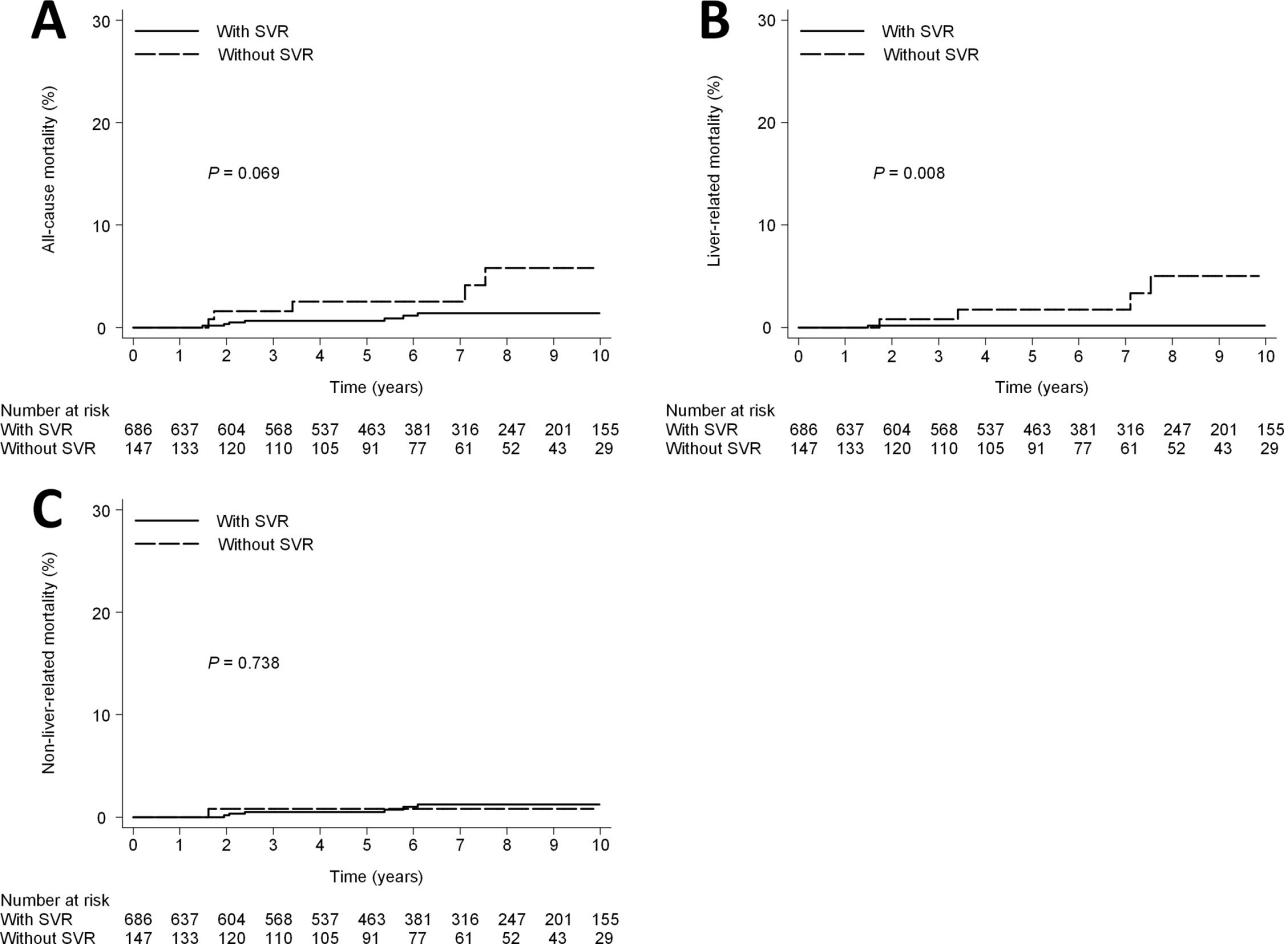
Mortality according to SVR in mild fibrotic patients. (A) all-cause mortality (10- year mortality rate, 1.5% vs 5.8%; HR, 0.36; 95% CI, 0.12–1.1; P = 0.07). (B) liver-related mortality (10- year mortality rate, 0.2% vs 5.0%; HR, 0.01; 95% CI, 0.02–0.54; P = 0.008). (C) non-liver-related mortality (10- year mortality rate, 1.3% vs 0.8%; HR, 1.4; 95% CI, 0.18–11.11; P = 0.74).

**Fig 5:**
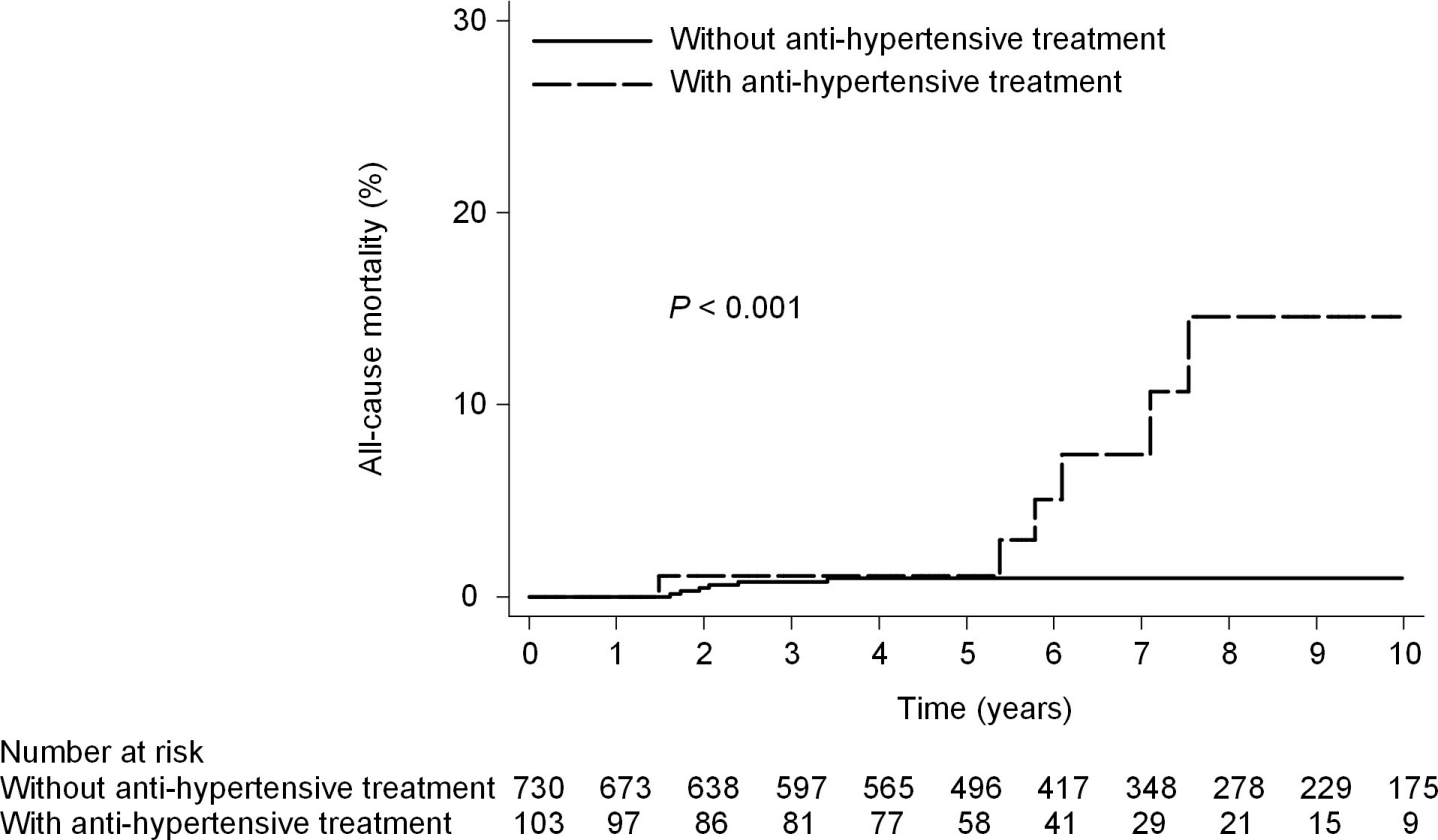
All-cause mortality according to anti-hypertensive treatment. (10- year mortality rate, 1.0% vs 14.6%; HR, 7.2; 95% CI, 2.5–21.3; P<0.001).

Seven patients with SVR and 5 without SVR died (10-year cumulative all-cause mortality rate, 1.4% [95% CI, 0.7%-3.0%] with SVR and 5.8% [95% CI, 2.3%-14.1%] without SVR; *P* = 0.069) (Fig 4A). Cox proportional hazards regression analysis showed that SVR was not associated with a statistically significant reduction in the overall hazard of death. The only one baseline factor found to be significantly associated with all-cause mortality in multivariate analysis was anti-hypertensive treatment (adjusted HR, 6.1; 95% CI, 1.7–22.5; P = 0.006) (Table 4). Six patients without anti-hypertensive treatment and 6 with anti-hypertensive treatment died (that is, the 10-year cumulative all-cause mortality rate was 1.0% [95% CI, 0.4%-2.1%] without anti-hypertensive treatment and 14.6% [95% CI, 6.4%-31.3%] with anti-hypertensive treatment; *P* < 0.001) (Fig 5).

**Table 4:** Features Associated With Mortality in Mild Fibrotic Patients (fibrosis score 0–2) (N = 833) According to Cox Proportional Hazards Model:Results of Multivariate Analyses.

			All-cause			Liver-related			Non-liver- related	
Variables	comparison	HR	95% CI	*P*	HR	95% CI	*P*	HR	95% CI	*P*
DM	Yes vs no	1.4	0.3–5.4	0.64	0.45	0.04–5.39	0.53	3.53	0.61–20.42	0.16
Anti-lipid treatment	Yes vs no	0.48	0.05–4.20	0.51	0.29	0.01–5.91	0.42	-		
Anti-hypertensive treatment	Yes vs no	6.12	1.66–22.54	0.006	12.34	1.4–108.54	0.02	2.78	0.49–15.89	0.25
SVR	Yes vs no	0.40	0.13–1.25	0.114	0.09	0.01–0.60	0.01	1.56	0.19–12.5	0.68
Age (yr)	≥60 vs <60	2.68	0.90–7.96	0.08	5.32	0.89–31.99	0.07	1.70	0.39–7.49	0.48
Gender	Male vs female	2.84	0.77–10.47	0.12	-			1.23	0.29–5.29	0.78
Obesity	BMI> vs ≤27 (kg/m^2^)	0.95	0.24–3.68	0.94	3.88	0.69–21.83	0.13	-		
Steatosis >5%	Yes vs no	1.88	0.60–5.89	0.28	2.39	0.4–14.14	0.34	0.89	0.17–4.53	0.89

DM, diabetes mellitus; SVR, sustained virological response; BMI, body mass index; Patients were categorized as obese (>27 kg/m^2^) according to the definition of the Health Promotion Administration of the Ministry of Health and Welfare in Taiwan [15].

### Liver-related mortality in mild fibrotic patients

**Fig 6:**
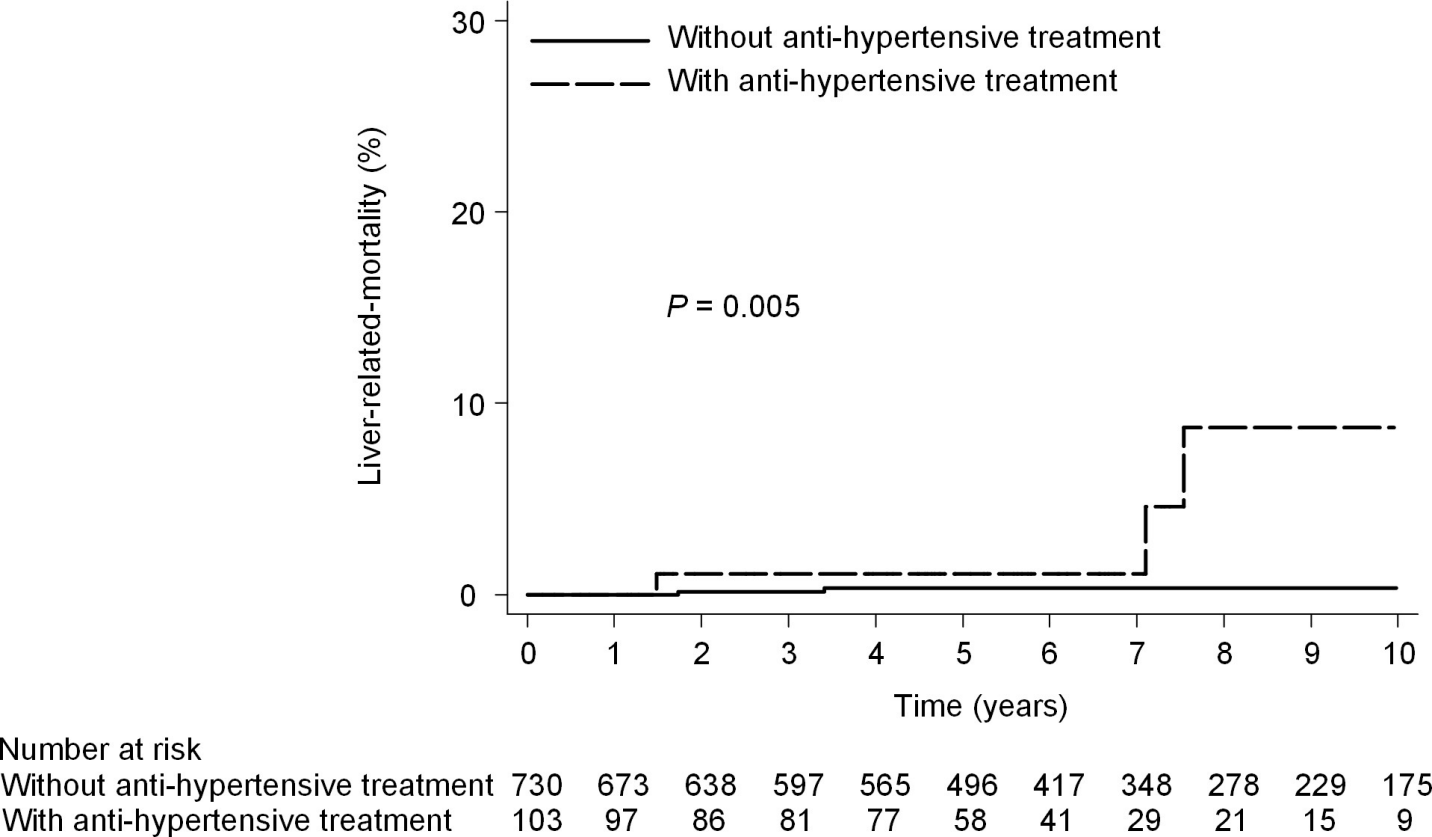
Liver-related mortality according to anti-hypertensive treatment. (10- year mortality rate, 0.3% vs 8.8%; HR, 10.3; 95% CI, 2.0–52.3; P = 0.005).

There was a significant difference in the cumulative 10-year liver-related mortality rate between patients with SVR (0.2%; 95% CI, 0.02%-1.1%) and those without SVR (5.0%; 95% CI, 1.8%-13.5%; *P* = 0.008) (Fig 4B). Cox proportional hazards regression analysis showed that SVR was associated with a statistically significant reduction in the hazard of liver-related mortality (adjusted HR, 0.09; 95% CI, 0.01–0.60; *P* = 0.013). The other baseline factor significantly associated with liver-related mortality in multivariate analysis was anti-hypertensive treatment (adjusted HR, 12.3; 95%CI, 1.4–108.5; P = 0.02) (Table 4). Two patients without anti-hypertensive treatment and 3 with anti-hypertensive treatment died (that is, the 10-year cumulative liver-related mortality rate was 0.3% [95% CI, 0.1%-1.3%] without anti-hypertensive treatment and 8.8% [95% CI, 2.6%-27.4%] with anti-hypertensive treatment; *P* = 0.005) (Fig 6).

Six patients with SVR and 1 without SVR died (10-year cumulative non-liver-related mortality rate, 1.3% [95% CI, 0.6%-2.8%] with SVR and 0.8% [95% CI, 0.1%-5.5%] without SVR; *P* = 0.738) (Fig 4C). Cox proportional hazards regression analysis showed that no baseline factors were significantly associated with non-liver-related mortality in multivariate analysis (Table 4).

### Baseline characteristics of patients with advanced fibrosis

There were 619 patients with advanced fibrosis, and 438 of these patients (70.8%) achieved SVR. As expected, the non-SVR patients, on average, were older, had a higher bilirubin level, and included larger proportions of patients with thrombocytopenia and genotype 1 than did the patients who achieved SVR (Table 5).

Causes of death are also shown in Table 5.

**Table 5:** Baseline Characteristics According to Treatment Response in advanced fibrotic patients.

Characteristics	Overall (N = 619)	With SVR (N = 438)	Without SVR (N = 181)	*P*
Age(yr)	57.4± 9.5	56.5 ± 9.6	59.6 ± 8.9	<0.001
Male	294 (47.5%)	222 (50.7%)	72 (39.8%)	0.01
Anti-hypertensive treatment	89 (14.4%)	62 (14.2%)	27 (14.9%)	0.81
Anti-lipid treatment	14 (2.3%)	10 (2.3%)	4 (2.2%)	0.96
DM	86 (13.9%)	63 (14.4%)	23 (12.7%)	0.58
BMI (kg/m^2^)				0.55
< 24	216 (39.3%)	159 (40.8%)	57 (35.9%)	
24–27	196 (35.7%)	135 (34.6%)	61 (38.4%)	
>27	137 (25.0%)	96 (24.6%)	41 (25.8%)	
Platelet count <150 (10^9^/L)	384 (62.3%)	248 (56.8%)	136 (76.0%)	<0.001
AST (IU/L)	112 (81–153)	111.5 (80–158)	112.5 (82–151)	1.0
ALT (IU/L)	149 (103–205)	153 (105–218)	130 (97–181)	0.04
Bilirubin (mg/dL)	1.05 ± 0.45	1.02 ± 0.41	1.14 ± 0.51	0.003
Necroinflammation score				0.45
<9	337 (54.5%)	234 (53.6%)	103 (56.9%)	
≥9	281 (45.5%)	203 (46.5%)	78 (43.1%)	
Genotype				<0.001
1	257 (45.9%)	151 (34.5%)	106 (58.6%)	
2	278 (49.6%)	238 (54.3%)	40 (22.1%)	
Mixed	25 (4.5%)	18 (4.1%)	7 (3.9%)	
others	59 (9.5%)	31 (7.1%)	28 (15.5%)	
Steatosis >5%	182 (29.4%)	142 (32.5%)	40 (22.1%)	0.03
Liver-related mortality				
		HCC, N = 8 Liver failure, N = 7 Liver transplantation, N = 1	HCC, N = 5 Liver failure, N = 12 Liver transplantation,N = 5	
Non–liver-related mortality				
		Bacterial infection,N = 3 Acute myocardial infarction, N = 1 Aortic dissection, N = 1 Chronic obstructive pulmonary disease, N = 1 Suicide, N = 1	Bacterial infection, N = 4 Buccal cancer, N = 1 Lung cancer, N = 1 Pancreatic cancer, N = 1 Gum cancer, N = 1 Renal failure, N = 1 Lymphoma, N = 1	

Data were expressed as mean ± SD or median (interquantile). DM, diabetes mellitus; BMI, body mass index; AST, Aspartate Aminotransferase; ALT, Alanine Aminotransferase; HCC, hepatocellular carcinoma. Patients were categorized as normal weight or underweight (<24 kg/m^2^), overweight (24–27 kg/m^2^), or obese (>27 kg/m^2^) according to the definition of the Health Promotion Administration of the Ministry of Health and Welfare in Taiwan [15].

### All-cause mortality in advanced fibrotic patients

**Fig 7:**
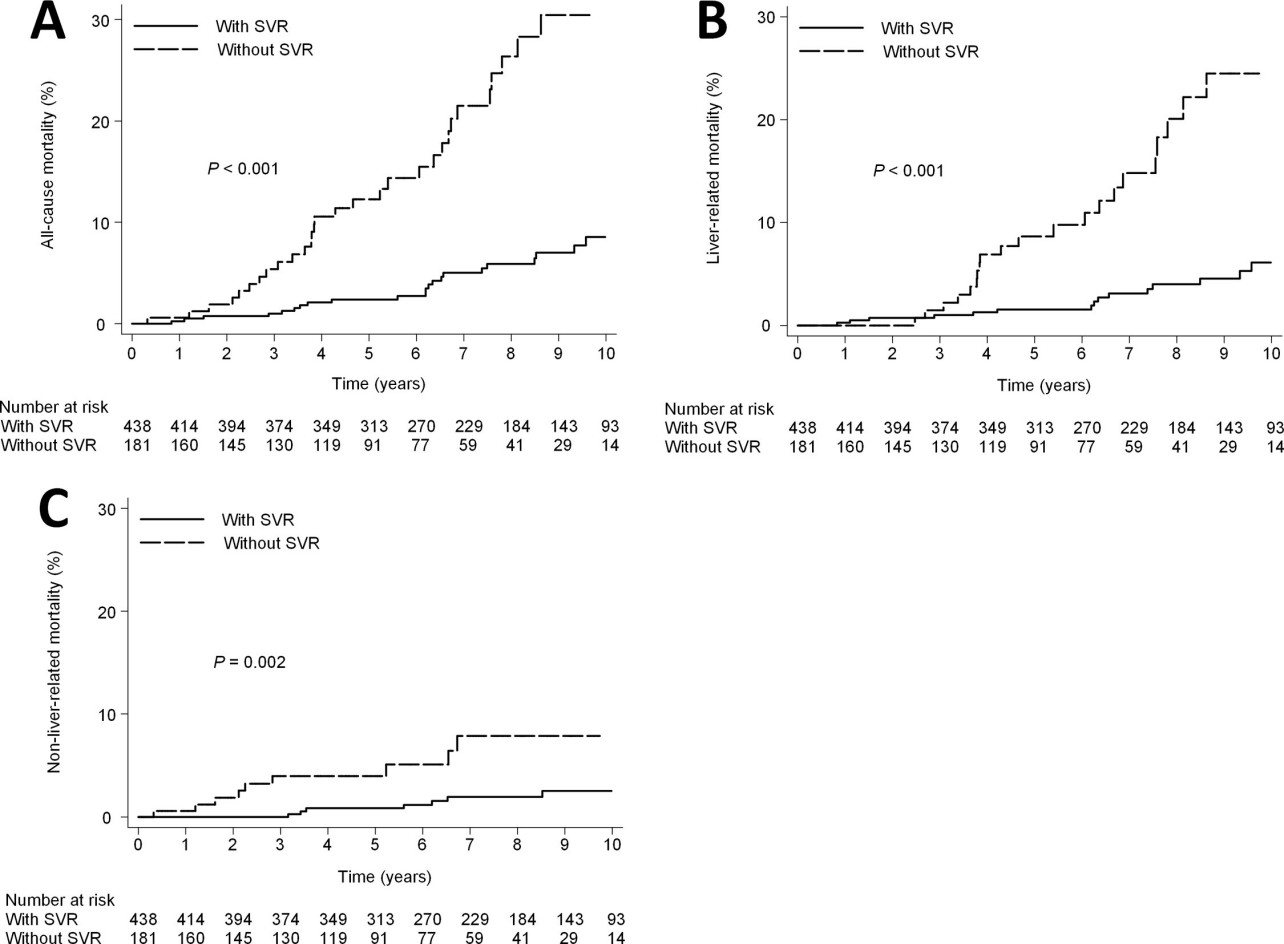
Mortality according to SVR in advanced fibrotic patients. (A) all-cause mortality (10- year mortality rate, 8.5% vs 30.4%; HR, 0.2; 95% CI, 0.12–0.35; P<0.001). (B) liver-related mortality (10- year mortality rate, 6.1% vs 24.5%; HR, 0.2; 95% CI, 0.10–0.38; P<0.001). (C) non-liver-related mortality (10- year mortality rate, 2.5% vs 7.9%; HR, 0.21; 95% CI, 0.08–0.56; P = 0.002).

There was a significant difference in the cumulative 10-year all-cause mortality rate between the patients with SVR (8.5%; 95% CI, 5.5%-13.1%) and those without SVR (30.4%; 95% CI, 21.5%-41.9%; *P*<0.001) (Fig 7A). Cox proportional hazards regression analysis showed that SVR was associated with a statistically significant reduction in the overall hazard of death (adjusted HR, 0.21; 95% CI, 0.12–0.37; *P*<0.001). The other baseline factor found to be significantly associated with all-cause mortality in multivariate analysis was male gender (Table 6).

**Table 6:** Features Associated With Mortality in Advanced Fibrotic Patients (fibrosis score 3–4) (N = 619) According to Cox Proportional Hazards Model:Results of Multivariate Analyses.

			All-cause			Liver-related			Non-liver-related	
Variables	comparison	HR	95% CI	*P*	HR	95% CI	*P*	HR	95% CI	*P*
DM	Yes vs no	1.35	0.68–2.68	0.40	0.85	0.32–2.22	0.73	2.55	0.86–7.57	0.09
Anti-lipid treatment	Yes vs no	-			-			-		
Anti-hypertensive treatment	Yes vs no	1.30	0.61–2.77	0.50	0.86	0.29–2.49	0.78	2.07	0.62–6.85	0.24
SVR	Yes vs no	0.21	0.12–0.37	<0.001	0.19	0.10–0.38	<0.001	0.26	0.10–0.71	0.009
Age (yr)	≥60 vs <60	1.44	0.83–2.48	0.20	1.08	0.56–2.11	0.81	2.53	0.9–7.13	0.08
Gender	Male vs female	1.74	1.01–2.98	0.05	1.56	0.81–2.98	0.18	2.24	0.82–6.09	0.11
Obesity	BMI> vs ≤27 (kg/m^2^)	1.10	0.57–2.13	0.78	1.11	0.5–2.45	0.80	1.05	0.31–3.51	0.94
Steatosis >5%	Yes vs no	0.58	0.29–1.17	0.13	0.79	0.37–1.72	0.56	0.18	0.02–1.35	0.10

DM, diabetes mellitus; SVR, sustained virological response; BMI, body mass index; Patients were categorized as obese (>27 kg/m^2^) according to the definition of the Health Promotion Administration of the Ministry of Health and Welfare in Taiwan [15].

### Liver-related mortality in advanced fibrotic patients

There was a significant difference in the cumulative 10-year liver-related mortality rate between the patients with SVR (6.1%; 95% CI, 3.6%-10.4%) and those without SVR (24.5%; 95% CI, 16.0%-34.5%; *P*<0.001) (Fig 7B). Cox proportional hazards regression analysis showed that SVR was associated with a statistically significant reduction in the hazard of liver-related mortality (adjusted HR, 0.19; 95% CI, 0.10–0.38; *P*<0.001) (Table 6).

### Non-liver-related mortality in advanced fibrotic patients

There was a significant difference in the cumulative 10-year non-liver-related mortality rate between the patients with SVR (2.5%; 95% CI, 1.2%-5.4%) and those without SVR (7.9%; 95% CI, 4.0%-15.2%; *P* = 0.002) (Fig 7C). Cox proportional hazards regression analysis showed that SVR was associated with a statistically significant reduction in the hazard of non-liver-related mortality (adjusted HR, 0.26; 95% CI, 0.10–0.71; *P* = 0.009) (Table 6).

### Bacterial infection viewed as liver-related mortality in patients with advanced fibrosis

Three patients with SVR died of bacterial infection, while 4 patients without SVR died of bacterial infection in patients with advanced fibrosis. If we consider bacterial infection as liver-related mortality, Cox proportional hazards regression analysis showed that SVR was associated with a statistically significant reduction in the hazard of liver-related-mortality (adjusted HR, 0.21; 95% CI, 0.11–0.38; *P*<0.001) and non-liver-related mortality (adjusted HR, 0.25; 95% CI, 0.07–0.95; *P* = 0.04) (Table 7).

**Table 7:** Features Associated With Mortality in Advanced Fibrotic Patients (fibrosis score 3–4, N = 619) According to Cox Proportional Hazards Model:Results of Multivariate Analyses (If we consider bacterial infection as liver-related mortality).

Variables	comparison	Liver Related			Non-Liver-Related		
		HR	95% CI	*P*	HR	95% CI	*P*
DM	Yes vs no	0.85	0.35–2.05	0.72	4.46	1.08–18.43	0.04
Anti-lipid treatment	Yes vs no	-			-		
Anti-hypertensive treatment	Yes vs no	0.86	0.33–2.24	0.75	3.40	0.76–15.29	0.11
SVR	Yes vs no	0.21	0.11–0.38	<0.001	0.25	0.07–0.95	0.04
Age (yr)	≥60 vs <60	1.18	0.64–2.18	0.59	3.05	0.73–12.7	0.13
Gender	Male vs female	1.65	0.91–3	0.10	2.19	0.58–8.24	0.24
Obesity	BMI> vs ≤27 (kg/m^2^)	1.33	0.66–2.68	0.43	0.33	0.04–2.9	0.32
Steatosis >5%	Yes vs no	0.62	0.29–1.32	0.21	0.36	0.04–3.04	0.35

DM, diabetes mellitus; SVR, sustained virological response; BMI, body mass index. Patients were categorized as obese (>27 kg/m^2^) according to the definition of the Health Promotion Administration of the Ministry of Health and Welfare in Taiwan [15].

### Whether metabolic risk factors were associated with mortality of all patients with SVR?

Cox proportional hazards regression analysis showed that anti-hypertensive treatment was associated with a statistically significant increased in the hazard of all-cause mortality (adjusted HR, 3.1; 95% CI, 1.27–7.51; *P* = 0.01) in all patients with SVR.

**Fig 8:**
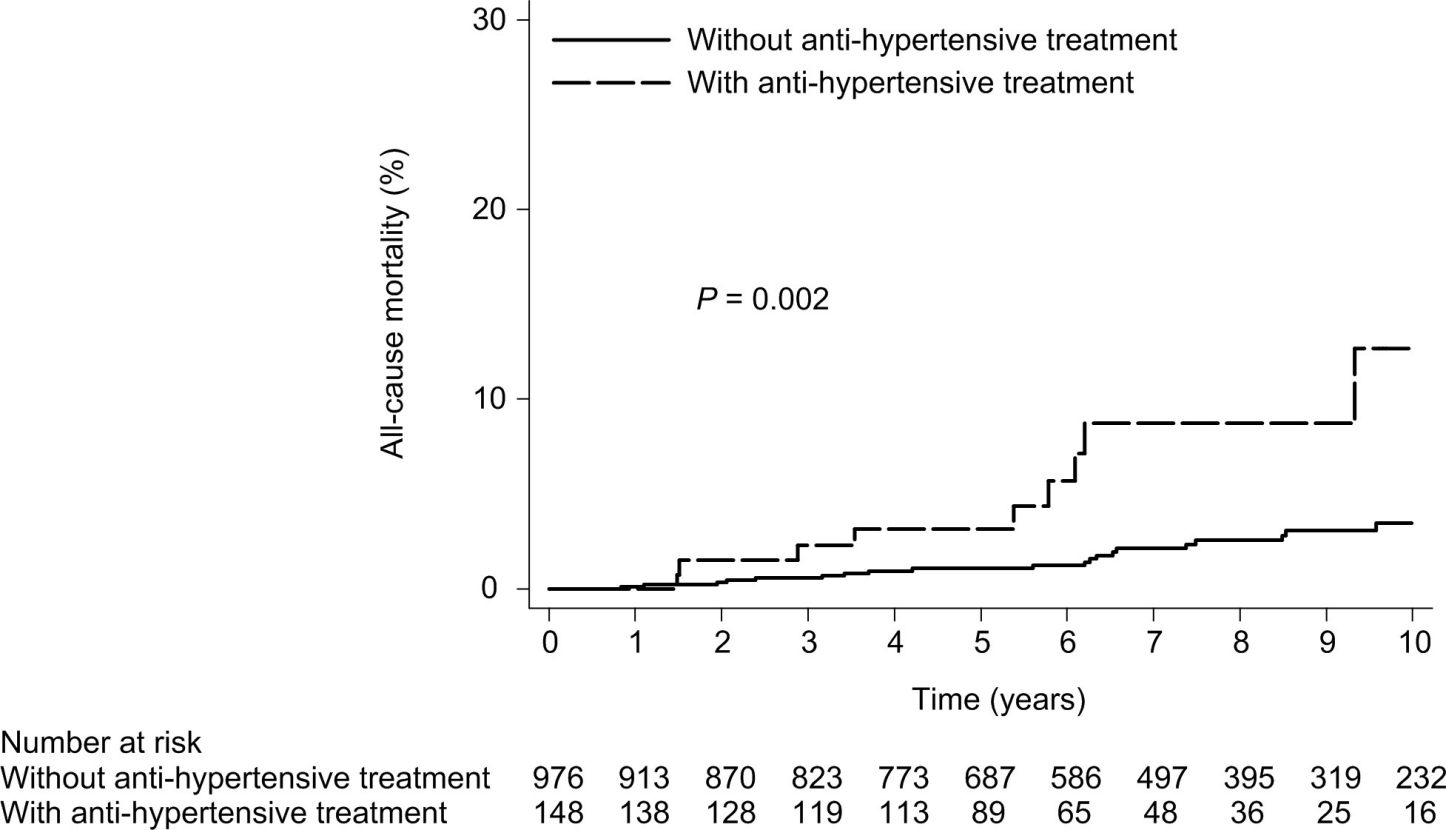
All-cause mortality according to anti-hypertensive treatment in all patients with SVR. (10- year mortality rate, with vs. without anti-hypertensive treatment: 12.68% vs. 3.46%, HR = 3.42, 95% CI = 1.58–7.44, *P* = 0.002).

Nine patients with anti-hypertensive treatment and 20 patients without anti-hypertensive treatment died (that is, the 10-year cumulative all-cause mortality rate was 12.68% [95% CI, 5.87%-26.23%] with anti-hypertensive treatment and 3.46% [95% CI, 2.17%-5.48%] without anti-hypertensive treatment; *P* = 0.002) (Fig 8). Metabolic risk factors were not associated with liver-related and non-liver-related mortality in all patients with SVR (Table 8).

**Table 8:** Features Associated With Mortality In All Patients with SVR (N = 1124) According to Cox Proportional Hazards Model:Results of Multivariate Analyses.

Variables	Comparison	All-cause			Liver-related			Non-liver-related		
		HR	95% CI	*P*	HR	95% CI	*P*	HR	95% CI	*P*
DM	Yes vs no	1.52	0.6–3.87	0.38	1.12	0.29–4.27	0.87	2.03	0.52–7.86	0.31
Anti-lipid treatment	Yes vs no									
Anti-hypertensive treatment	Yes vs no	3.08	1.27–7.51	0.01	2.64	0.77–9.08	0.13	3.52	0.94–13.21	0.06
Fibrosis score	3–4 vs 0–2	3.64	1.66–7.99	0.001	11.85	2.69–52.23	0.001	1.36	0.47–3.98	0.57
Age (yr)	≥60 vs <60	2.34	1.14–4.78	0.02	1.81	0.7–4.69	0.22	3.13	1.04–9.42	0.04
Sex	Male vs female	2.18	1.03–4.64	0.04	2.09	0.78–5.6	0.15	2.20	0.68–7.11	0.19
Obesity	BMI> vs ≤27 (kg/m^2^)	0.51	0.17–1.53	0.23	0.42	0.09–1.96	0.27	0.63	0.13–3.08	0.57
Steatosis >5%	Yes vs no	0.58	0.25–1.36	0.21	0.76	0.27–2.19	0.62	0.38	0.08–1.74	0.21

DM, diabetes mellitus; SVR, sustained virological response; BMI, body mass index. Patients were categorized as obese (>27 kg/m^2^) according to the definition of the Health Promotion Administration of the Ministry of Health and Welfare in Taiwan [15].

### Whether metabolic risk factors were associated with mortality of mild fibrotic patients with SVR?

**Fig 9:**
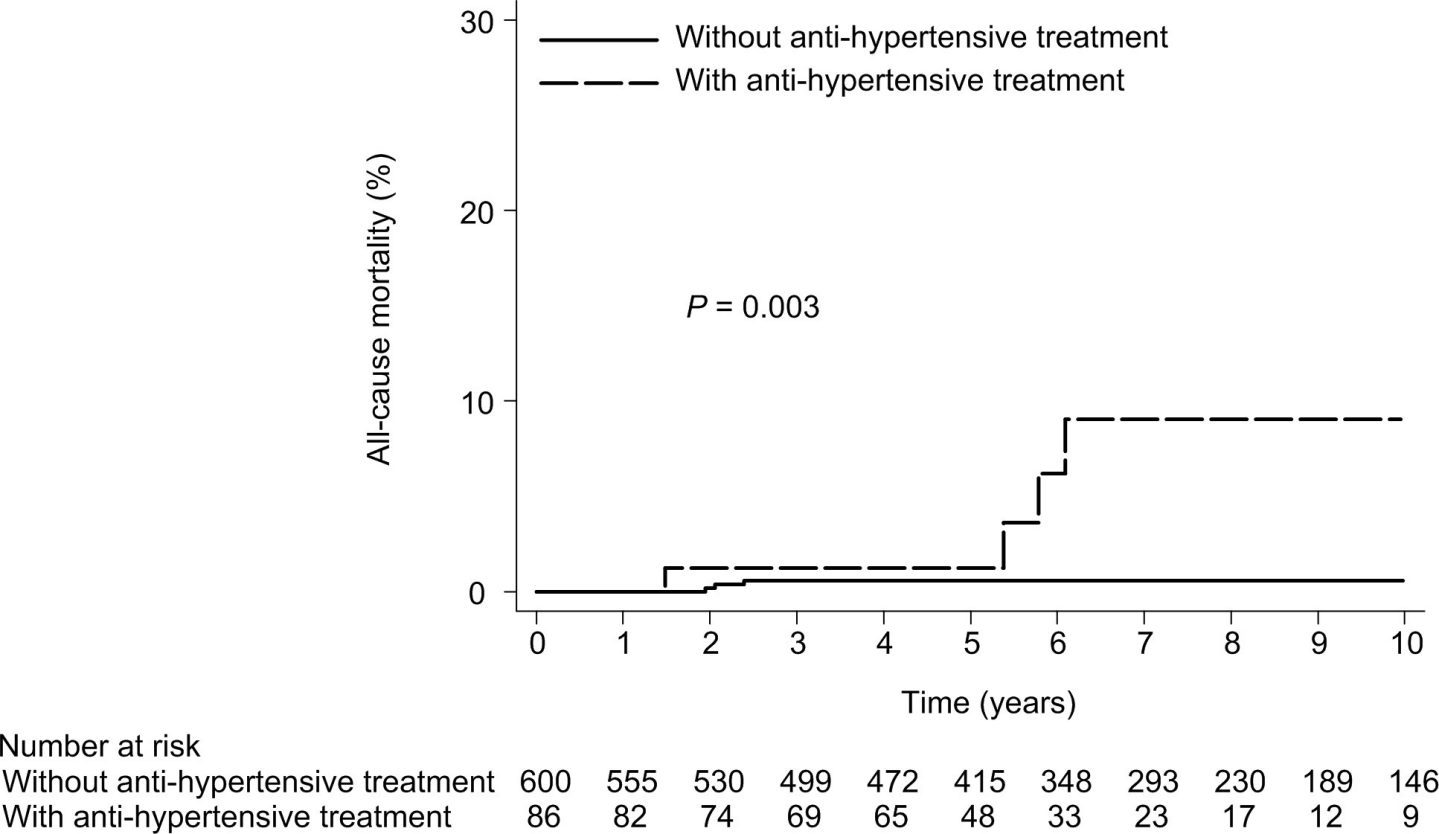
All-cause mortality according to anti-hypertensive treatment in mild fibrotic patients with SVR (10-year mortality rate, with vs. without anti-hypertensive treatment: 9.05% vs. 0.57%, HR = 7.72, 95% CI = 2.03–29.42, *P* = 0.003).

Cox proportional hazards regression analysis showed that anti-hypertensive treatment was associated with a statistically significant increased in the hazard of all-cause mortality (adjusted HR, 5.8; 95% CI, 1.14–29.07; *P* = 0.03) in mil fibrotic patients with SVR. Four patients with anti-hypertensive treatment and 3 patients without anti-hypertensive treatment died (that is, the 10-year cumulative all-cause mortality rate was 9.05% [95% CI, 3.36%-23.13%] with anti-hypertensive treatment and 0.57% [95% CI, 0.18%-1.76%] without anti-hypertensive treatment; *P* = 0.003) (Fig 9).

Metabolic risk factors were not associated with liver-related and non-liver-related mortality in mild fibrotic patients with SVR (Table 9).

**Table 9:** Features Associated With Mortality In Mild Fibrotic Patients (fibrosis score 0–2) with SVR (N = 686) According to Cox Proportional Hazards Model:Results of Multivariate Analyses.

Variables	Comparison	All-cause			Liver-related			Non-liver-related		
		HR	95% CI	*P*	HR	95% CI	*P*	HR	95% CI	*P*
DM	Yes vs no	2.20	0.41–11.88	0.36				4.02	0.59–27.27	0.15
Anti-lipid treatment	Yes vs no									
Anti-hypertensive treatment	Yes vs no	5.76	1.14–29.07	0.03	7.06	0.37–134.1	0.19	3.05	0.46–20.14	0.25
Age (yr)	≥60 vs <60	3.86	0.99–15.04	0.05				2.29	0.49–10.78	0.29
Sex	Male vs female	3.07	0.61–15.39	0.17				1.96	0.37–10.45	0.43
Obesity	BMI> vs ≤27 (kg/m^2^)	0.43	0.05–3.85	0.45	2.36	0.11–48.63	0.58			
Steatosis >5%	Yes vs no	1.75	0.42–7.35	0.44	1.91	0.1–35.45	0.67	1.09	0.2–5.91	0.92

DM, diabetes mellitus; SVR, sustained virological response; BMI, body mass index. Patients were categorized as obese (>27 kg/m^2^) according to the definition of the Health Promotion Administration of the Ministry of Health and Welfare in Taiwan [15].

### Whether metabolic risk factors were associated with mortality of advanced fibrotic patients with SVR?

Cox proportional hazards regression analysis showed that metabolic risk factors were not associated with all-cause, liver-related and non-liver-related mortality in advanced fibrotic patients with SVR (Table 10).

**Table 10:** Features Associated With Mortality In Advanced Fibrotic patients (fibrosis score 3–4) with SVR (N = 438) According to Cox Proportional Hazards Model:Results of Multivariate Analyses.

Variables	Comparison	All-cause			Liver-related			Non-liver-related		
		HR	95% CI	*P*	HR	95% CI	*P*	HR	95% CI	*P*
DM	Yes vs no	1.22	0.38–3.89	0.73	1.39	0.36–5.39	0.63	0.68	0.07–6.49	0.74
Anti-lipid treatment	Yes vs no									
Anti-hypertensive treatment	Yes vs no	2.36	0.77–7.26	0.13	2.31	0.58–9.3	0.24	2.23	0.35–14.23	0.40
Age (yr)	≥60 vs <60	1.93	0.83–4.46	0.13	1.24	0.44–3.5	0.68	5.39	1.02–28.45	0.05
Sex	Male vs female	2.05	0.86–4.89	0.11	1.75	0.63–4.85	0.28	2.86	0.55–14.97	0.21
Obesity	BMI> vs ≤27 (kg/m^2^)	0.54	0.15–1.97	0.35	0.23	0.03–1.87	0.17	1.40	0.24–8.4	0.71
Steatosis >5%	Yes vs no	0.40	0.13–1.18	0.10	0.66	0.21–2.08	0.47			** **

DM, diabetes mellitus; SVR, sustained virological response; BMI, body mass index. Patients were categorized as obese (>27 kg/m^2^) according to the definition of the Health Promotion Administration of the Ministry of Health and Welfare in Taiwan [15].

## Discussion

In the present large-sample study of patients with baseline histological evaluation of liver fibrosis, SVR was associated with lower rates of all-cause, liver-related and non-liver-related mortality in all patients and in the patients with advanced fibrosis, in addition to being associated with a lower rate of liver-related mortality in the mild fibrosis patients. All the patients included in this study were treated with an interferon-based regimen between 1999 and 2011 because DAAs only became available in November 2014 in Taiwan. The results of this study showed that SVR reduced the risk of liver-related mortality irrespective of fibrosis stage. Furthermore, SVR reduced the risk of all-cause mortality in all patients and the patients with advanced fibrosis, effects which have been well documented in previous studies [3].

The most important finding of this study was that SVR also reduced the risk of non-liver-related mortality in all patients and in the patients with advanced fibrosis. A previous study reported that SVR reduced the risk of non-liver-related mortality in patients with cirrhosis [4]. Nahon, et al. conducted a prospective study which enrolled 1323 liver biopsy-proven cirrhotic CHC patients. In that study, the patients with SVR had a lower risk of cardiovascular events and bacterial infections, which may have translated to a reduced risk of non-liver-related mortality in that cohort [4].

Among the 17 instances of non-liver-related mortality in advanced fibrotic patients in our study, 3 patients in the SVR group died due to bacterial infections, while 4 patients in the non-SVR group died due to bacterial infections. No patients in the SVR group died due to extrahepatic cancers, while 5 patients in the non-SVR group died due to extrahepatic cancers. We did not have data regarding the cumulative incidence of bacterial infections and extrahepatic cancers during the follow-up period in our study. Therefore, we can only speculate that SVR decreased the risks of bacterial infections and extrahepatic cancers in the patients with advanced fibrosis in our study.

On the other hand, in the group of patients with mild fibrosis, one patient in the non-SVR group died due to extrahepatic cancer, while 3 patients in the SVR group died due to extrahepatic cancers. One possible explanation for this interesting finding is significantly higher use of statins therapy among non-SVR patients with mild fibrosis, statins therapy could reduce not only prevalence of hepatic, but also extrahepatic cancers in CHC patients [16]. Finally, patients who achieved SVR have a substantially reduced risk for extrahepatic cancers in all patients in our study (relative risk = 0.15). A recent review article reported that HCV might be associated with an increased risk of extrahepatic cancers [17]. Chronic HCV has been shown to affect cellular signaling pathways promoting cancer formation as well as inhibiting tumor suppressor genes [18]. A previous study reported that SVR did not influence the occurrence of extrahepatic malignancies [4], but more evidence is needed to further explore this issue.

Bacterial infections are one of the important causes of mortality in patients with cirrhosis. Bacterial infections develop as a consequence of immune dysfunction that occurs progressively during the course of cirrhosis [4]. SVR over the long term might be associated with cirrhosis regression [19], but also with disruption of a vicious cycle triggered by cirrhosis-related bacterial infections [20]. It is customary to view bacterial infections as an extrahepatic complication, with the exception of spontaneous bacterial peritonitis, which was classified in the present study as liver-related. However, SVR also reduced the risk of liver-related and non-liver-related mortality in the patients with advanced fibrosis if we viewed deaths due to bacterial infections in general as a form of liver-related mortality.

In the present study, only 4 of the patients died of cardiovascular disease, including one patient with heart failure, two patients with acute myocardial infarction, and one patient with aortic dissection. All of these patients achieved SVR. Fatal cardiovascular events were surprisingly found only in SVR patients. We could speculate that CHC patients have rarely atherogenic dyslipidemia [21] and CHC patients with SVR after IFN-based therapy had significant increase of total cholesterol and low density lipoprotein (LDL)-cholesterol [22]. Cardiovascular death, particularly among CHC patients without SVR, may be reduced by high liver-related mortality, mortality for extrahepatic cancers and bacterial infection.

With regard to metabolic risk factors, DM was found to be associated with an increased risk of non-liver-related mortality among all patients in this study. Anti-hypertensive treatment was found to be associated with an increased risk of all-cause mortality in all patients, as well as increased risks of all-cause and liver-related mortality in the patients with mild fibrosis. A previous population-based study reported that DM increased the risk of all-cause mortality in CHC patients [23]. DM is a well-known risk factor for cardiovascular disease and cancers [24]. In addition, infectious diseases are more frequent and/or serious in patients with DM [25], which potentially increases their mortality and which could explain the aforementioned finding.

According to the European Association for the Study of the Liver (EASL) guidelines, non-alcoholic steatohepatitis (NASH) patients with fibrosis associated with hypertension should receive closer monitoring because of a higher risk of disease progression [10]. Hypertension is a well-known risk factor for cardiovascular disease, and this could explain the association of anti-hypertensive treatment with all-cause and liver-related mortality in the patients with mild fibrosis in this study. A population-based study reported that hypertension was positively associated with liver-related mortality in a CHC cohort. In that study, hypertension was defined as a systolic blood pressure (BP) of ≥140 mm Hg and diastolic BP of ≥90 mm Hg or a history of anti-hypertensive treatment; the study did not mention, however, whether or not the CHC cohort ever underwent interferon-based treatment [5]. In the present study, meanwhile, we did not record the blood pressure of each patient. All the patients did undergo interferon-based treatment, however, which may have suppress hepatocarcinogenesis irrespective of SVR [26]. Therefore, liver-related mortality should be lower in our cohort compared with the cohort from the previous population-based study [5].

In the era of interferon-free therapy, most of CHC patients might achieve SVR. Whether metabolic risk factors are associated with mortality of CHC patients with SVR is an important issue. Anti-hypertensive treatment was found to be associated with an increased risk of all-cause mortality in all patients with SVR, as well as increased risk of all-cause mortality in mild fibrotic patients with SVR in our study. A recent study also reported that hypertension was associated with an increased risk of all-cause mortality in those who achieved SVR after DAAs therapy [27].

The strengths of this study were the baseline histological evaluation of liver fibrosis. There were limitations in this study. First, this study was limited by short-term follow up. Second, this study investigated patients treated at a single institution and was retrospective nature. Therefore, it was possible that the lost-to-follow-up patients affected the mortality incidence. Third, tobacco consumption, alcohol intake and injection drug use, which are important causes of death, were not routinely recorded. We assume, however, that the prevalence of injection drug use in our cohort was low due to a previous study that reported that the most common genotypes among injection drug users in Taiwan were genotypes 1a, 6 and 3 [28], whereas these genotypes were rare in our cohort (34 patients with genotype 1a, 7 patients with genotype 3, and 8 patients with genotype 6). Fourth, our study did not involve a central pathologist for the interpretation of liver histology. Thus, there were interobserver discrepancies in the assessments of hepatic fibrosis. Fifth, waist circumference and blood pressure were not routinely recorded for each patient, while fasting blood sugar and lipid levels were also not routinely tested in each patient. Therefore, we could not examine the impacts of truncal obesity and metabolic syndrome (both of which are important risk factors for cardiovascular events and liver disease progression [5, 29, 30]) on mortality. Sixth, some people may argue that evaluating data on mortality in a population of patients with chronic HCV infection treated with interferon-based therapies at a time when DAAs-based interferon free regimens have totally supplanted worldwide, the use of the older regimens may be pointless. However, limited studies investigated the impact of DAAs therapy on survival [27, 31]. Two recent observational cohort analysis used data from the the Department of Veterans Affairs’ Clinical Case Registry for HCV showed that SVR was independently associated with reduced risk of all-cause mortality compared to non-SVR in HCV-infected veterans, irrespective of fibrotic stage [27, 31]. Aforementioned studies only investigated the impact of DAAs therapy on all-caused mortality and did not analyzed whether DAAs therapy improved non-liver related mortality, which is the most important point of our study. Furthermore, the limitations of these studies including the cohorts were overwhelmingly male (96–97%) [27, 31] and short-term follow up (a mean follow-up period of 1.6 years) [27].

In conclusion, the results of this study indicated that SVR to interferon-based treatment was associated with lower rates of all-cause, liver-related, and non-liver-related mortality in all patients and the patients with advanced fibrosis, as well as with a lower rate of liver-related mortality in the mild fibrosis patients.

Further studies enrolling DAAs-treated patients who undergo long-term follow-up would help us to understand whether SVR after DAAs treatment improves survival among chronic HCV-infected patients, including older patients who had poorer tolerability to IFN-based therapy [32–34] and patients with severe comorbidities which are contraindications for IFN-based therapy [11]. Recent studies reported that patients with old age, comorbidities or poor liver functional reserve are at higher risk of short-term mortality after DAAs therapy [35, 36]. These data reinforce the need of studies evaluating the real impact of DAAs therapy on clinical outcomes in elderly or severe comorbidities patients who could not benefit from DAAs therapy. DM was associated with an increased risk of non-liver-related mortality in all patients in this study. Furthermore, anti-hypertensive treatment was associated with an increased risk of all-cause mortality in all patients, as well as increased risks of all-cause mortality and liver-related mortality in the patients with mild fibrosis. A future population-based study enrolling more patients who undergo long-term follow-up, including the use of non-invasive tests to evaluate liver fibrosis, would be able to clarify the association between hypertension and liver-related mortality in CHC patients.

## Supporting information

S1 Data (XLSX)
